# Effective monitoring of online AI decision-making algorithms in just-in-time adaptive interventions

**DOI:** 10.1038/s41746-026-02669-4

**Published:** 2026-05-04

**Authors:** Anna L. Trella, Susobhan Ghosh, Erin E. Bonar, Lara Coughlin, Finale Doshi-Velez, Yongyi Guo, Pei-Yao Hung, Inbal Nahum-Shani, Vivek Shetty, Maureen Walton, Iris Yan, Kelly W. Zhang, Susan A. Murphy

**Affiliations:** 1 Department of Computer Science, Harvard University, Cambridge, MA USA; 2 Department of Psychiatry, University of Michigan, Ann Arbor, MI USA; 3 Department of Statistics, University of Wisconsin-Madison, Madison, WI USA; 4 Institute for Social Research, University of Michigan, Ann Arbor, MI USA; 5 School of Dentistry & Engineering, University of California, Los Angeles, Los Angeles, CA USA; 6 Mathematics Department, Imperial College London, South Kensington, London UK; 7 Department of Statistics, Harvard University, Cambridge, MA USA

**Keywords:** Computational biology and bioinformatics, Engineering, Health care, Mathematics and computing, Medical research

## Abstract

Monitoring just-in-time adaptive interventions (JITAIs) is important both during trialing and when the intervention is deployed in a broader healthcare program. While there is increasing interest in using artificial intelligence (AI) algorithms in JITAIs, these algorithms introduce additional complexity that requires additional monitoring. In this paper, we provide guidelines for monitoring online AI decision-making algorithms. Our guidelines include: (1) identifying potential issues, categorizing them by severity (red, yellow, and green), and (2) developing fallback methods (pre-specified procedures that are executed when an issue occurs). To make ideas concrete, we discuss algorithm monitoring systems in two case studies. In both, the monitoring systems detected real-time issues, and fallback methods both safeguarded participants and ensured quality data for post-deployment data analysis to further refine the JITAI. These guidelines and findings give teams the confidence to include online AI decision-making algorithms in JITAIs.

## Introduction

Just-in-Time Adaptive Interventions (JITAIs)^1^ are increasingly common digital health interventions^2–6^ and have significant potential to improve health outcomes^7–11^. Effective monitoring of software systems supporting JITAIs, and more broadly digital interventions, is essential for maintaining intervention quality, reliability and data integrity by mitigating risks, identifying issues early, and allowing timely corrective actions. For example, networking issues could prevent cloud computing services from delivering the intervention to smart devices (e.g. phones, wearables). Monitoring systems are well-established in the field of clinical trials; in this case, monitoring is used to safeguard participants, ensure data integrity, and to ensure that the trial is compliant with the approved protocol and regulatory requirements^12^. In clinical trials, examples of monitoring systems include Data and Safety Monitoring Boards (DSMB) that oversee participant safety and data quality^13^, Source Data Verification^14^ and statistical monitoring^15^to ensure data quality, and risk-based monitoring^16^ tools to ensure participant safety. There are also frameworks for monitoring implementation fidelity^17,18^, the degree at which an intervention is delivered as intended. These frameworks^19–22^ help accurately interpret the intervention’s effectiveness by ensuring any modifications or exceptions are properly documented.

There is increasing interest to include online AI decision-making algorithms in JITAIs^8,23–28^. These algorithms refine JITAI decision rules that control intervention delivery (e.g., which intervention option to deliver or how often an intervention gets delivered) to maximize their overall effectiveness^29–31^. *While many of the aforementioned monitoring systems are useful for monitoring JITAIs, additional monitoring is required when JITAIs include an online AI decision-making algorithm*. This additional monitoring is required because the online AI decision-making algorithm updates the JITAI decision rules. If the online AI decision-making algorithm functions incorrectly, it detracts from the intended deployment of the JITAI, and in turn could compromise individuals’ experience. This may further reduce engagement with and efficacy of the intervention, lower the quality of data used for analyses^32^ that guide JITAI development, and ultimately jeopardize future developments and use of these promising algorithms.

This paper provides: (1) guidelines for monitoring online AI decision-making algorithms in JITAIs and (2) two case studies in which monitoring systems were deployed. Our guidelines include: (1) identifying potential issues, categorizing them by severity (red, yellow, and green), and (2) developing fallback methods (pre-specified procedures which are executed when an issue occurs). The two case studies described in the paper (Oralytics and MiWaves) involve JITAIs in which an online AI decision-making algorithm is used to update the JITAI’s decision rules. These case studies offer in-depth examples and findings gained from implementing real-time monitoring systems for such algorithms.

## Results

### Setting and constraints

Constraints can impact the structure of the monitoring system. For example, the constraints in Oralytics were: (1) a lack of control over data processing and storage due to dependence on an external sensing system and (2) a short time frame for developing the monitoring system. In contrast, MiWaves had greater control over data processing and storage and fewer time constraints. These differing settings and constraints led to the development of different monitoring systems for Oralytics and MiWaves.

Due to different time constraints, the two monitoring systems made different trade-offs between coverage of issues and efficiency in implementation. Since Oralytics had limited time to develop the monitoring system, the system specified broad categories of issues (Supplementary Table 1 in Supplementary Section D). MiWaves had more time to develop the monitoring system and specified more detailed issues (Supplementary Table 2 in Supplementary Section E). The development of the two monitoring systems reflects different trade-offs between implementation speed and diagnostic granularity. Due to tight timelines, Oralytics employed broad checks, such as a single alert for failed communication with the backend controller. While this shortened the initial development time to meet the trial start date, it resulted in higher maintenance overhead; the development team spent more time manually triaging and investigating the root causes of any issues that occurred during deployment. Conversely, the MiWaves team leveraged insights from the Oralytics deployment to implement more granular checks, such as verifying specific authorization headers in backend controller requests. This proactive design required greater upfront engineering effort, but provided richer context when issues occurred, resulting in a more streamlined triage process and faster resolution.

Another difference between the Oralytics and MiWaves monitoring systems stemmed from their level of control over their data pipelines. Oralytics depended on an external system for pre-processed sensor data (e.g., brushing duration from an electronic toothbrush) which was then stored in a database managed by the backend controller (see Supplementary Section F for the database schema). Because Oralytics had no control over the initial data capture, its monitoring system focused on validating the reliability of this external source. This involved, for example, checking if the data was in the correct format or if the data fields were non-empty. In contrast, MiWaves had direct control over its entire pipeline, as it managed data capture, local storage, and processing (see Supplementary Section G and Supplementary Tables 3–9 for MiWaves database schema). Consequently, its monitoring system was not focused on validating a third-party sensor, but rather on ensuring the reliability of its own internal processes. This not only included monitoring for communication issues with the backend controller, but also verifying its own data capture and processing steps. The differing circumstances between Oralytics and MiWaves show how different constraints influence the design of monitoring systems in JITAIs. See Supplementary Section D for further details on development effort of the monitoring systems for Oralytics and MiWaves.

### Monitoring interface and operational workflow

Here we describe: (1) the concrete implementation of the monitoring interface with the underlying technologies used, and (2) how the system was used in practice by staff during deployment.

**Fig. 1:**
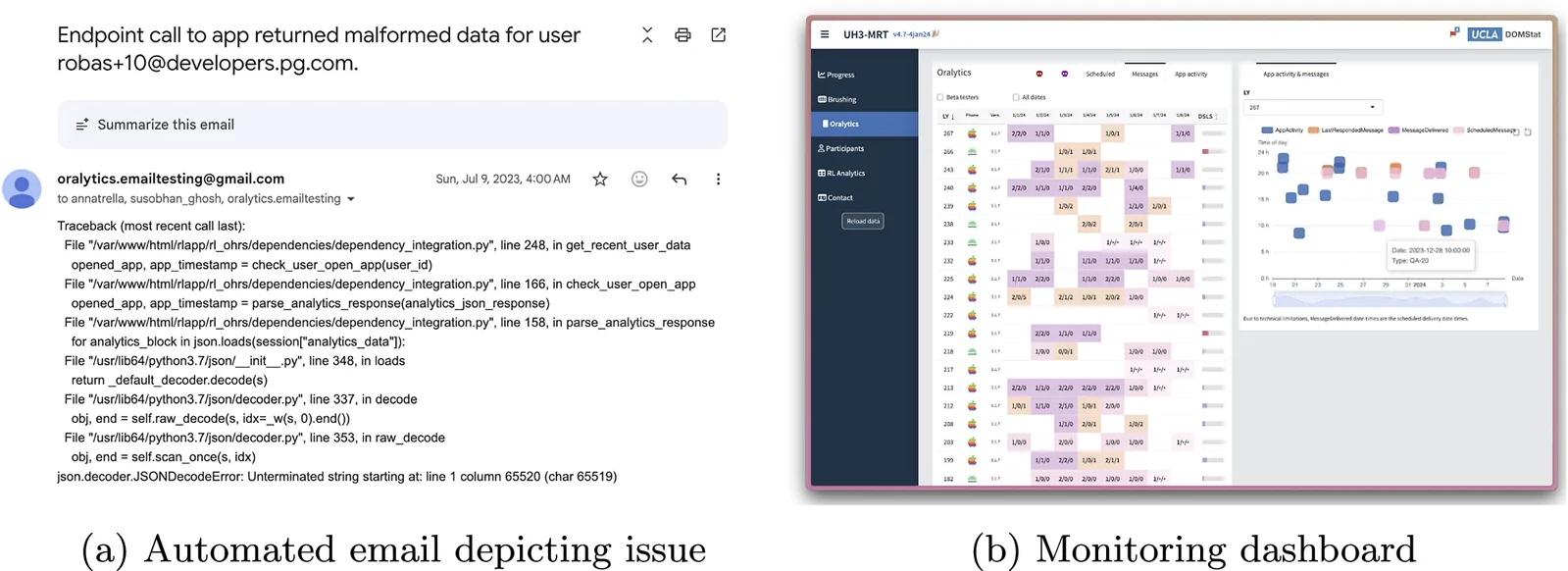
Oralytics Monitoring Interfaces. For Oralytics, the two interfaces for interacting with the system are (**a**) automated emails sent when an issue is detected and (**b**) a browser-based dashboard. The automated email shown here was triggered by a yellow-severity issue for malformed data and the dashboard displays key metrics such as scheduled messages and participant app activity.

For Oralytics, monitoring was conducted through two primary interfaces (Fig. 1): automated email alerts implemented using Flask-Mail and a browser-based dashboard built in Shiny (RStudio). The majority of monitoring functionality was implemented through automated email alerts, which were triggered only when an issue was detected. These emails provided a description of the error and indicated whether a fallback procedure had been executed. In addition, selected errors and operational metrics were integrated into the dashboard. The dashboard was manually reviewed by staff on a daily basis and aggregated key metrics from multiple data sources, including the RL algorithm and the mobile application. Examples of displayed metrics include the number of messages scheduled per participant, as well as message scheduling successes and errors.

**Fig. 2:**
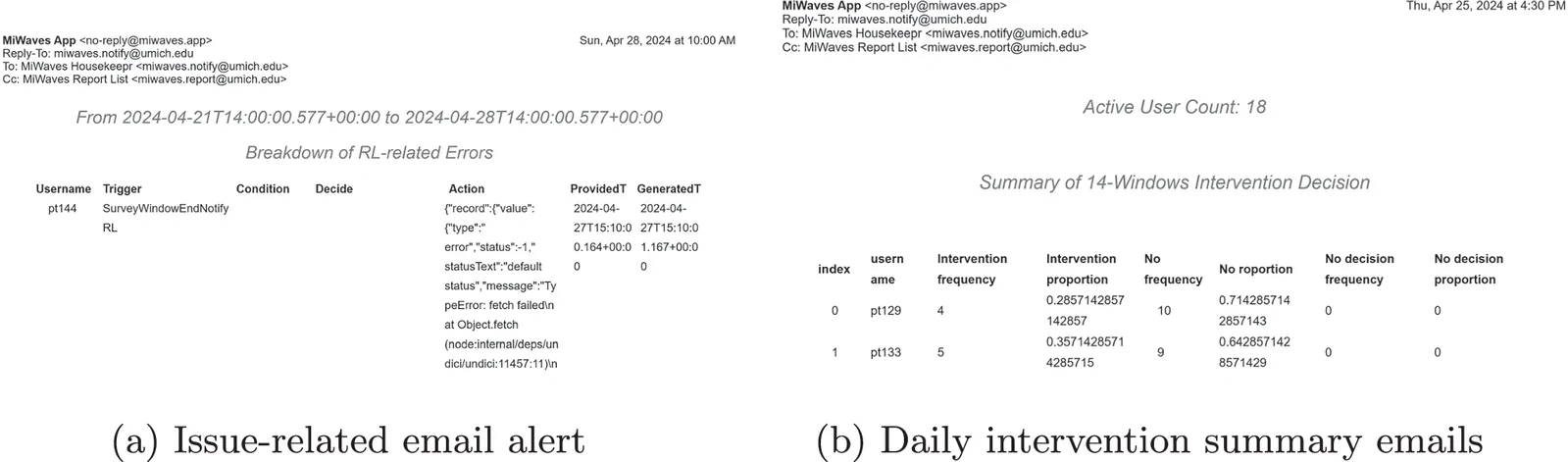
MiWaves Monitoring Interfaces. For MiWaves, the two interfaces for interacting with the system are (**a**) automated emails sent when an issue is detected and (**b**) a daily summary email of the intervention decisions taken by the algorithm. **a**The automated email shown was triggered by a yellow-severity issue which occured while uploading data to the RL algorithm at the end of a decision time. **b** The daily summary email showed a summary of what intervention decisions were taken for individuals (without named identifiers) on a rolling 14-decision time window (1 week), and included statistics like frequency and proportion of times they were sent an intervention and not sent an intervention.

For MiWaves, the monitoring system was set up using Python Flask and utilized automated emails set up using Node.js. The monitoring system utilized these automated emails to alert staff if any issues were detected, and if any fallback methods were executed (see Fig. 2a for one such email). Moreover, an automated email (with a different email subject) summarizing the intervention decisions taken by the algorithm in a 7-day rolling window was sent daily (example in Fig. 2b). The staff monitored their email inbox daily for any automated emails that were raised due to issues.

In both trials, the standard operational workflow for responding to issues caught by the monitoring system was the same. Upon detecting an issue (either manually or automatically), the workflow consisted of alerting the RL algorithm development team, triaging and understanding the issue, taking steps to solve the issue, and properly documenting key details (see Section 7 for key examples).

### Fallback methods

To clarify the Oralytics fallback methods presented later, we first discuss the *backup intervention schedule*. A critical Oralytics design decision was to construct a backup intervention schedule, a schedule of assigned intervention prompts, each morning for all remaining decision points for participants in the 70-day trial. This means that while the updated JITAI decision rules were used to output action-selection probabilities and hence select actions (i.e., intervention options) for a particular day’s decision points, a backup intervention schedule was also formed for potential use at each of the individual’s remaining decision points. See Supplementary Section B.4 for full details on the backup intervention schedule. MiWaves did not employ a backup intervention schedule.

Oralytics employed a two-layer fallback method for determining which action to deliver and a separate method for updating: (i) if there were any communication issues between the backend controller and the app, intervention prompt from the most recent backup intervention schedule saved on the Oralytics app was delivered; and (ii) if there were any issues in constructing the backup intervention schedule, then the binary action for each decision point was assigned with 0.5 probability (iii) if issues arose with pulling or storing data (e.g.,malformed data or recent data was not available), then the algorithm stored the data point, but did not update the JITAI decision rules using that data point.

We now describe the fallback methods for MiWaves. If the JITAI decision rules failed to assign actions to participants, then the MiWaves backend controller assigned binary actions to participants instead (i.e. intervention prompt versus no prompt were sent with 0.5 probability). If the RL algorithm could not update the JITAI decision rules, the most recent JITAI decision rules were used to assign interventions. This ensured that even when the RL algorithm failed to update JITAI decision rules, nonsensical decision rules were not used to assign interventions. This difference in fallback design was driven by the system architecture; the Oralytics app’s ability to function offline with its backup schedule was a safeguard against the external sensor’s data pipeline, whereas MiWaves’ fully-controlled pipeline allowed for a centralized fallback logic in the backend controller.

In the Oralytics trial, all three fallback methods were triggered in response to yellow severity issues such as failing to obtain necessary data or the RL algorithm crashing^33^. In MiWaves, fallback methods were triggered in response to three yellow severity issues when the RL algorithm was unresponsive or had crashed. The fact that fallback methods were triggered in both case studies highlight the importance of planning ahead for issues so that the digital health intervention system functions appropriately. See Supplementary Section J for a quantitative report on all fallback methods executed in both trials.

### Issues and Severity

In this section, we highlight key examples and insights of issues that occurred for each severity level. For a comprehensive list of potential issues that were checked from each trial, please refer to Supplementary Table 1 (in Supplementary Section D) and Supplementary Table 2 (in Supplementary Section E). Furthermore, for a quantitative report of issues that occurred during both trials refer to Supplementary Section J.

1. *Red Severity*: Both Oralytics and MiWaves checked if: (i) the RL algorithm’s updated JITAI decision rules assigned an unreasonable number of intervention prompts (less than the minimum amount or more than the maximum amount) and (ii) action selection probabilities returned by the updated JITAI decision rules for any participant at any given decision point stayed within a bounded limit (see Supplementary Section B.3). Due to time constraints on software development, Oralytics monitoring staff manually checked for red severity issues, whereas in the MiWaves trial, this check was automated in the software and an automated email would be sent to the staff and all relevant development teams if such issues were detected. The automation in MiWaves allowed for more comprehensive checks. For example, MiWaves included checks for an unreasonable number of assigned prompts (i) at any decision point as well as for individual participants over seven days. Although these types of checks were implemented, issues in categories (i) and (ii) were never detected in either Oralytics nor MiWaves.

During the MiWaves clinical trial, the development teams added an additional red severity issue to detect if the cloud computer had low available memory. This precaution was taken after two incidents occurred during the trial where the available memory was too low, causing the backend controller to malfunction, and RL algorithm along with the database to crash. Low available memory on the cloud computer is a red severity issue because it can cause the backend controller to crash, preventing interventions from being sent to participants. In order to preemptively mitigate the impact of this issue, the cloud computer’s memory was increased, and automated email checks were designed to notify the backend controller development team when the cloud computer exceeded a specified memory usage threshold. After implementation, these checks were triggered once subsequently in the MiWaves trial, which prompted the backend development team to increase the available memory once again.

2. *Yellow Severity*: For detecting yellow severity issues, Oralytics implemented checks such as ensuring requests for sensor data (originally from external source but processed and made available to the RL algorithm by the backend controller) were successful, verifying that the sensor data was correctly formatted, and ensuring that the RL algorithm can properly read and write data to its own database.

For example, during the Oralytics trial, the RL algorithm crashed and was unavailable on two separate occasions. This meant that the JITAI decision rules were also unavailable, and unable to output actions. However, because of fallback method (i), participants were assigned interventions from the most recent backup intervention schedule saved on their Oralytics app. The RL development team restarted the system and the RL algorithm (along with the JITAI decision rules) became available again the next day. For additional yellow issues that occurred during the trial, see Supplementary Section A.1.

In MiWaves, the majority of the yellow severity issues focused on verifying the functionality of the RL algorithm and its components, and checking for any associated issues. For example, a yellow severity issue was encountered on the first day of the MiWavestrial, when certain participants were removed after the research staff discovered that they were fraudulent (see Supplementary Section H). However, this information was not communicated to the RL algorithm. As a result, there was a discrepancy between the actual number of active participants in the trial and the number recorded as active by the RL algorithm. This mismatch caused the RL algorithm to use the wrong JITAI decision rule for a participant (participant A was incorrectly provided participant B’s intervention option). Within one day, the backend controller and app development teams addressed the issue by implementing a fix (in both the backend controller and the RL algorithm) and restarting the RL algorithm to prevent similar scenarios in the future. The fix involved adding a check to communicate about active participants, and henceforth the RL algorithm maintained the correct set of active participants in the trial. All yellow severity issues encountered during the MiWaves trial are detailed in Supplementary Section A.2.

3. *Green Severity*: Unlike red and yellow severity issues, which require a technological fix, green severity issues are resolved by completing documentation essential for adjusting post-deployment data analyses. In both Oralytics and MiWaves, a majority of green severity issues were resolved by documenting the details and resolutions of red and yellow severity issues (e.g., if a red severity issue that was technically fixed, but not yet documented, it would remain as a green severity issue until the documentation was complete). However, other documentation tasks, such as logging information on crashes, resets, or reboots of the RL algorithm, or problems with other system components (e.g., backend controller) outside of the RL algorithm, were also tracked as green severity issues. The documentation details the timestamps, the number of decision points and participants impacted, what fallback method was used (for yellow severity issues), and how the relevant development team fixed the issue.

Early during the Oralytics trial, intervention staff noticed that very few participants had intervention prompts scheduled on their apps. Upon investigation, the RL algorithm and the associated JITAI decision rules were functioning correctly, but the backend controller failed to wait long enough to gather the backup intervention schedules for all participants, resulting in blank schedules being sent to some participants. The issue was resolved the next business day, and the RL development team documented the incident and the affected participants and decision points.

In MiWaves, an issue occurred when the cloud computer ran out of available memory, causing the RL algorithm to crash abruptly during its daily update of JITAI decision rules. Since the update was not marked as successful, the RL algorithm continued to use pre-crash JITAI decision rules upon rebooting. The JITAI decision rules were successfully updated the following day, with the timestamp recorded in the database.

## Discussion

Algorithm monitoring systems are essential to support the use of online AI decision-making algorithms in JITAIs, both within clinical trials and broader health care systems. While monitoring is well-established in traditional clinical trials, which rely on Data and Safety Monitoring Boards (DSMB)^13^ and risk-based monitoring^16^ to safeguard participants, the introduction of online AI decision-making algorithms requires additional real-time monitoring of such algorithms. To that end, we provided guidelines for algorithm monitoring and described the use of algorithm monitoring systems in two case studies where online RL algorithms dynamically adjusted the likelihood of intervention delivery based on incoming data. Designing and implementing a monitoring system is a worthwhile investment. As demonstrated in Section Results, the absence of a monitoring system could lead to critical issues, such as individuals not receiving any intervention prompts or the use of biased data in data analyses. These issues represent a breakdown in implementation fidelity^17,18^, a core concept in existing frameworks^19–22^ that we have here adapted for the unique complexities of autonomous, data-driven, online AI decision-making. Although our paper focused on online AI decision-making algorithms (especially RL algorithms abundant in JITAIs), we expect many findings are applicable to a variety of online learning algorithms, such as those used for fine-tuning prediction algorithms (e.g., predicting stress) in JITAIs. We hope these monitoring guidelines empower teams to confidently incorporate the innovative benefits that online AI decision-making algorithms offer in JITAIs.

While we provide practical examples that support current state-of-the-art online decision-making algorithms used in JITAIs, the issues, severity levels, and fallback methods described here are not exhaustive. As algorithms in this space become more advanced, this framework may need to be expanded to include additional issues and fallback methods. Specifically, the emerging use of Large Language Models (LLMs) in high-stakes decision-making introduces novel monitoring challenges^34^ that require specialized reliability architectures^35^ and alignment with evolving risk management profiles^36^. Future iterations of these guidelines must adapt to the non-deterministic nature of such generative systems to ensure participant safety and intervention fidelity remain robust as the AI decision making system increases in complexity.

## Methods

To illustrate the guidelines, we draw upon insights from two case studies of JITAIs that deployed online AI decision-making algorithms to refine JITAI decision rules. These JITAI decision rules control intervention delivery (e.g., send or not send an intervention prompt). These JITAIs exemplify both practical successes achieved and practical difficulties encountered in monitoring real-world online decision-making algorithms. Case study 1 (details in Section Case Study 1: Oralytics) describes a clinical trial of the Oralytics intervention^29,33,37–40^ which focuses on improving tooth-brushing behaviors for participants at risk for dental disease. Case study 2 (details in Section Case Study 2: MiWaves) describes a clinical trial of the MiWaves intervention^41–43^ that aims to reduce cannabis use amongst emerging adults (ages 18-25). In both of these JITAIs, the intervention prompts encourage engagement in healthy behaviors.

### Online AI decision-making preliminaries

This section provides a brief overview of online AI decision-making algorithms in JITAIs. Online decision-making algorithms are different from offline decision-making algorithms. In offline decision making, an existing data set is used to refine JITAI decision rules for use in future deployments of the JITAI. Online decision-making algorithms are used to refine JITAI decision rules for the individuals experiencing the intervention. This latter approach is used when societal shifts, population changes, or substantial heterogeneity in individual responsivity may warrant different JITAI decision rules than those used in prior deployments.

A majority of online AI decision-making algorithms utilized in JITAIs are from the area of reinforcement learning (RL)^44^, a branch of machine learning focused on methods for optimizing decision rules. In RL, the decision rules are called *policies* and the optimization goal is to maximize *rewards*. Generally rewards are a function of proximaloutcomes coded so that high values are preferable (e.g., brushing quality, an individual’s app engagement). In RL, the intervention options are called *actions* (e.g., send or do not send an intervention prompt). Note that the RL algorithm’s policy may output a single, specific action to take, or may output a distribution over actions (for instance, an action-selection probability of sending an intervention prompt). In RL, the *state* includes potential tailoring variables(e.g., current mood, time of day, recent brushing quality, recent dose of interventions). The *decision points* (e.g., one hour before an individual usually brushes their teeth) are the times at which RL uses the policy and current state to select an action. For simplicity, we refer to the online AI decision-making algorithms in both case studies as RL algorithms for the rest of the paper.

In Oralytics and MiWaves, both RL algorithms use a *model* of the reward that is used to provide a prediction of how the reward will change at each decision point based on the individual’s state and the selected action at that decision point. Further, at pre-specified *update times* (e.g. nightly, weekly), the RL algorithms update estimates of the model parameters and measures of confidence in the estimates of the parameters using prior interaction history (i.e., individual’s state at prior decision points, prior actions and prior rewards). Using the updated model, the RL algorithms update the JITAI decision rules. The RL algorithms use these updated decision rules at each decision point to output action-selection probabilities (the probability to send an intervention prompt) based on the individual’s current state, and an action is then selected according to those probabilities.

### General software framework

**Fig. 3:**
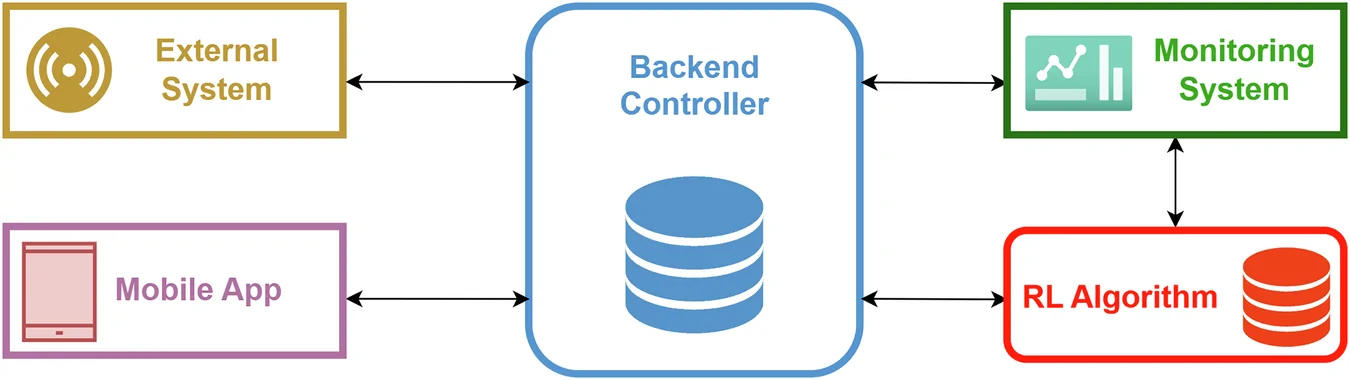
Overview of the software components in the intervention system for both case studies. The backend controller acts as the central coordinator and interacts with the mobile app, the RL algorithm, and external systems (i.e. third party services not controlled by the app, backend or RL development team---like proprietary toothbrush sensors). The monitoring system pulls data from the backend controller and the RL algorithm to facilitate both automated and human monitoring of the intervention performance and operational status in real-time.

Multiple software components are included in the intervention system. Figure 3 provides the components used in both Oralytics and MiWaves. The *backend controller* (maintained by the *backend controller development team*) acts as the central coordinator of the system, and manages the communication between external systems (if any), the RL algorithm (maintained by the *RL development team*), and the smartphone mobile app (maintained by the *app development team*). *External systems* provide data (e.g., sensor data needed for state and reward) to the backend controller from third-party sources (e.g., a proprietary toothbrush sensor data provider). The backend controller processes this data and sends the data to the *RL algorithm*. In both Oralytics and MiWaves, the actions are binary: send an intervention prompt versus not (see below for more detail). The backend controller obtains actions from the RL algorithm and provides them to a *mobile app* that delivers engagement prompts based on the actions (i.e., selected intervention options). A *monitoring system* pulls data from all components for both automated and staff-monitoring. Staff-monitoring involves the use of a dashboard to monitor the implementation fidelity of the JITAI and operational status in real-time. A component of this monitoring system is the algorithm monitoring system (in Elements of the Algorithm Monitoring System section). In this paper, we are primarily focusing on the algorithm monitoring system. With the exception of the mobile app, all other components of the software system (including the RL algorithm) used in each case study were deployed on a *cloud computer*.

### Case Study 1: Oralytics

**Fig. 4:**
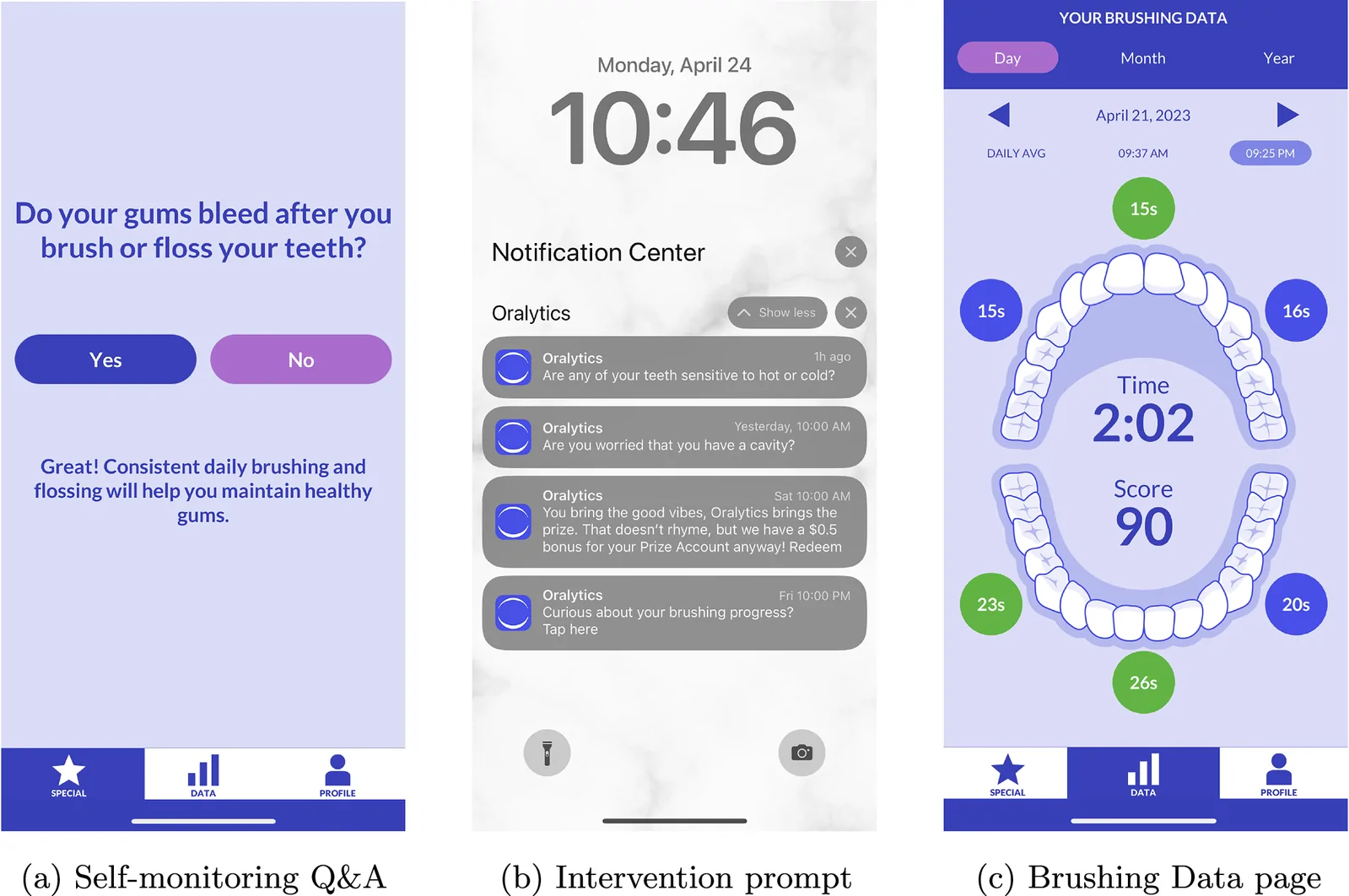
Screenshots from the Oralytics app. **a** an example self-monitoring Q& A message, (**b**) example intervention prompt to promote engagement, and (**c**) the `Your Brushing Data' page, which displays an individual’s brushing duration, quadrant distribution, and score.

The Oralytics JITAI is designed to improve the proximal outcome of oral self-care behaviors (OSCB) for individuals at risk of dental disease. Individuals receive a commercially-available electric toothbrush with Bluetooth connectivity and integrated sensors that monitors brushing behavior in real-time and automatically transmits this data to a proprietary data provider. They can self-monitor and view their brushing behavior in the Oralytics app. To facilitate high-quality OSCB, individuals receive intervention prompts via push notifications from the Oralytics app (Fig. 4). The intervention prompts contain content grounded in psychological research known to support behavior change^39^.

Oralytics uses an RL algorithm^33,38,40^ to update its JITAI decision rules which decide whether or not to deliver a prompt at two decision points per day, one hour prior to an individual’s usual morning and evening brushing windows. The reward for the Oralytics RL algorithm is a function of the OSCB, measured in seconds. To determine the individual’s state, the RL algorithm considers factors such as time of day (morning or evening), recent brushing behavior, the number of prompts recently delivered, and recent app engagement (see Supplementary Section B.2 for exact definition of state). The RL algorithm updates the JITAI decision rules on a weekly cadence. See Supplementary Section B for more information on the Oralytics RL algorithm.

The Oralytics case study was a one-armclinical trial with *N* = 79 participants recruited from UCLA dental clinics in Los Angeles, where each participant was provided the Oralytics JITAI. The trial ran from September 2023 to July 2024, with each participant being in the trial for 70 days. The study protocol and consent procedures have been approved by the University of California, Los Angeles Institutional Review Board (IRB#21-001471) and the clinical trial was registered on ClinicalTrials.gov (NCT05624489). Please refer to^37,39^ for a detailed description of the Oralyticstrial.

### Case study 2: MiWaves

**Fig. 5:**
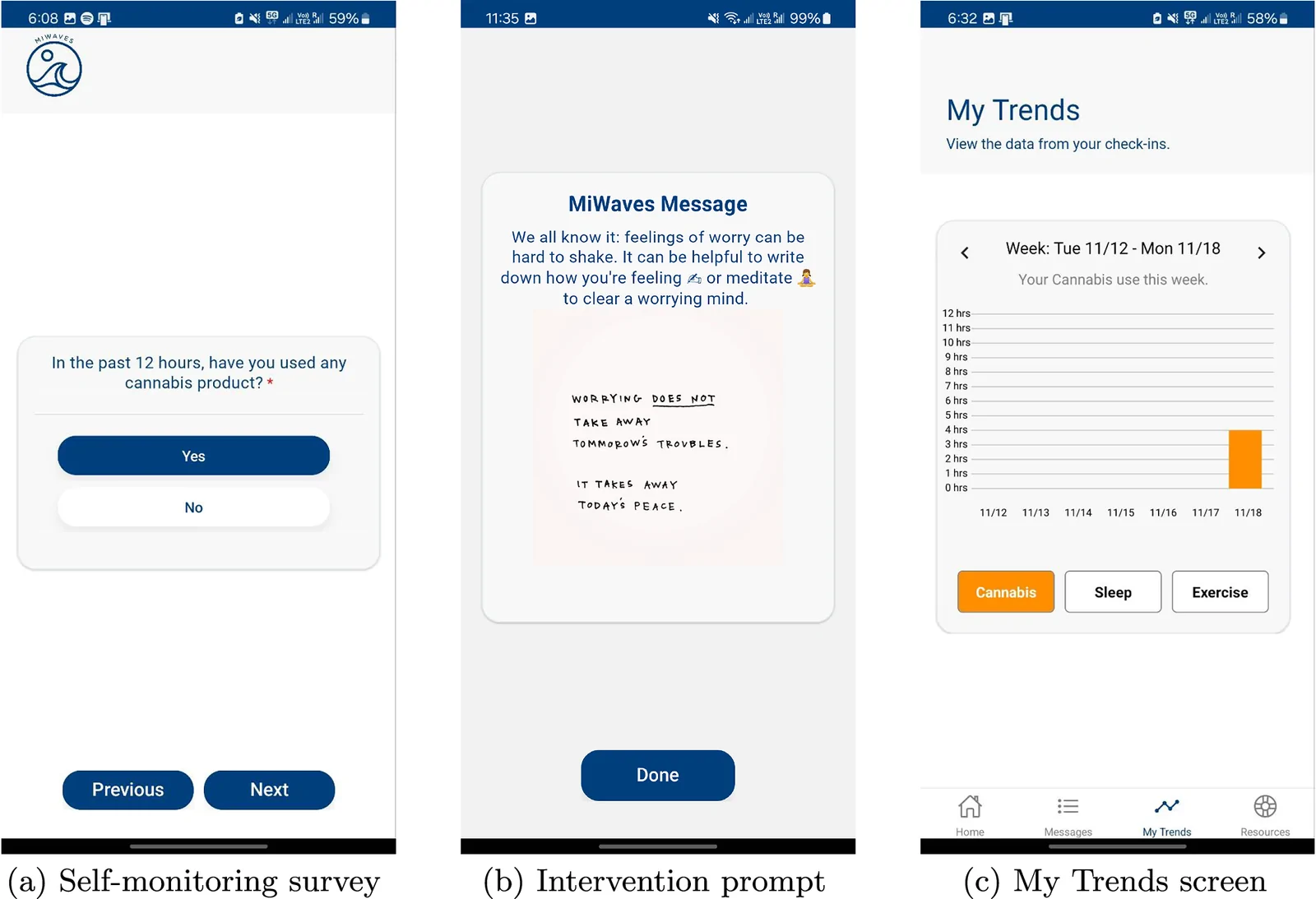
Screenshots from the MiWaves app. **a** Self-monitoring check-in screen – one of the questions prompting participants to report cannabis use in the past 12 hours, **b** an example of an intervention message providing motivational content to encourage reflection and mindfulness, and (**c**) the `My Trends' screen, which displays participants' self-reported trends in cannabis use, sleep, and exercise over the past week, helping them reflect on their lifestyle habits.

The MiWaves JITAI is designed to help reduce cannabis use among emerging adults (EAs)^45^. Individuals are asked to self-monitor (their sleep, cannabis use, alchohol use, etc.; see Fig. 5a) twice daily (morning and evening) using the MiWaves mobile app, and intervention prompts (see Fig. 5b) are delivered to individuals upon completion or expiration of self-monitoring via the MiWaves mobile app. The intervention prompts contain behavior change strategies, such as strategies to reduce negative and enhance positive affect, increase cannabis-free activities, and enhance future/goal-directed thinking. The MiWaves app also allowed individuals to visualize their weekly self-reported data using bar plots (see Fig. 5c).

MiWaves uses an RL algorithm^42^ to update its JITAI decision rules which decide, twice daily, whether or not to deliver an intervention prompt containing a behavior change strategy. To do so, the MiWaves RL algorithm utilizes data coming from the self-monitoring and mobile app usage ofparticipating individuals. The reward for the RL algorithm was the participant’s engagement level with the app and intervention prompts. The RL algorithm utilizes an individual’s time-of-day, recently reported cannabis use behavior and recent app and intervention engagement as state (see Supplementary Section C.1 for exact definition of state). The RL algorithm autonomously updates the JITAI decision rules on a daily cadence. See Supplementary Section C and^43^ for more information on the MiWaves RL algorithm.

The MiWaves case study was a one-arm clinical trial with *N* = 122 participants recruited across the U.S. through social media, in which all participants were provided the MiWaves JITAI. The trial ran from March 2024 to May 2024, with each participant being in the trial for 30 days. The study protocol and consent procedures have been approved by the University of Michigan Institutional Review Board (IRB#HUM00222194) and the trial was registered on ClinicalTrials.gov (NCT05824754). Please refer to^41^ for more information.

### Why monitor?

Online RL algorithms generally include two processes: (1) learning and (2) action selection. Learning involves modeling how individuals’ state and reward vary with actions and updating the estimates of the model’s parameters (repeatedly estimated) as data accrues. Notice that updating the model also updates the policy (i.e. JITAI decision rules) which specify which of the actions (i.e. intervention options) to deliver based on the individual’s current state. Action selection involves the algorithm using the updated policy to select actions at each decision point. To ensure these two processes work correctly, it’s essential to accurately collect and transmit current state and reward data to the RL algorithm. If this data is inaccurate or missing, these two processes might cause issues that could compromise individuals’ experience and the utility of data for post-deployment data analyses (i.e., further refinement of the JITAI). For example, communication issues between the RL algorithm and the backend controller can lead to individuals receiving too many or too few prompts, or prompts at the incorrect times. Or the software system may fail to save data needed to conduct high-quality post-deployment data analyses.

Autonomously monitoring the fidelity of RL algorithms for intervention delivery is important for detecting, alerting, and preventing issues such as those presented above. A monitoring system prevents such issues and detects issues as soon as they arise. This allows the JITAI system to identify, triage, and resolve issues which (1) minimizes negative impacts on individuals and (2) reduces the burden on the staff who would otherwise need to manually monitor.

### Elements of the algorithm monitoring system

Tables 1 and 2 provide a summary of the guidelines for building a real-time monitoring system for an RL algorithm. These guidelines consists of: (1) issues and severity and (2) fallback methods. Both categories of guidelines work with each other to avoid issues that otherwise would have occurred. In the Results section, we provide concrete examples from Oralytics and MiWaves.

**Table 1:** Classification of algorithm monitoring issues by severity

Issue Severity	Description	Example	Action item
Red	Issues that compromise individuals’ experiences or the scientific utility of data	Algorithm assigns an unreasonable number of interventions per individual over a period of time	Urgent remediation required. Document, fix, and verify resolution immediately.
Yellow	Issues that compromise the ability of the algorithm to learn and select actions	Failure to obtain current state from the backend controller	Document and resolve. Urgency is secondary to red severity issues.
Green	Issues that need to be documented so that post-deployment data analyses can be properly adjusted	RL algorithm is repaired after it crashes, but the details of the repair or fallback method executed are not documented	Document the issue, and ensure all incident details are passed to the data analyst for post-deployment adjustments.

Potential issues are categorized into three levels of severity (red, yellow and green) based on their impact on participant experience, algorithm functionality and post-deployment data utility. Note that effective fallback methods (summarized in Table 2) may downgrade the operational severity of a red issue to yellow by safeguarding the participant experience in real-time. Also note that green severity issues do not require fallback methods.

**Table 2:** Summary of fallback methods for online AI Decision-making

Fallback Methods	Description	Example of Fallback
For Intervention Decisions	Automated procedures executed when action selection errors occur using the algorithm’s updated JITAI decision rules	The system defaults to a pre-defined backup schedule or assigns the intervention prompt with a fixed probability (e.g., *p* = 0.5).
For Learning & Updates	Procedures executed when the algorithm fails to update JITAI decision rules	The algorithm excludes corrupted data when updating JITAI decision rules, and if required, postpones the update and continues using the most recent JITAI decision rule until next update time.

These pre-specified procedures ensure baseline intervention delivery and system stability when technological issues prevent standard algorithm learning or action selection. Note that effective fallback methods may downgrade the operational severity of a red issue to yellow by safeguarding the participant experience in real-time. Also note that green severity issues do not require fallback methods.

#### Issues and severity

All development teams should begin by identifying and assigning severity to potential issues that could occur. This allows teams to prioritize addressing issues based on their severity. Once these issues are identified, an automated notification system (e.g., automated email) can alert the monitoring team when an issue occurs. We recommend categorizing issues into three severity levels:

1. *Red Severity (Safeguarding Individuals and Utility of Data)*. Red severity issues compromise individuals’ experiences or the utility of the data for further intervention refinement, and requires immediate attention. These issues should trigger an automated notification to the RL development team and should be prominently displayed on the monitoring dashboard for the monitoring team. Individuals’ experiences can be compromised if they receive an unreasonable amount of intervention prompts. This could result from communication errors between software components, invalid action selection by the RL algorithm’s updated JITAI decision rules, or in the case of a clinical trial, failing to recognize that a participant is no longer part of the trial. The scientific utility of the data could be compromised if the database fails to store necessary values because it timed-out, closed connections, or exceeded its storage capacity.
2. *Yellow Severity (Compromises Algorithm Functionality).* Yellow severity issues compromise the ability of the online RL algorithm to learn (update the JITAI decision rules) or select actions using the most up-to-date decision rules. We specifically define compromising learning in terms of technological failures (e.g., malformed inputs) rather than logical factors (e.g., bad initialization, distribution shifts, statistical efficiency). Importantly, compromising learning does not necessarily imply an immediate degradation in individuals’ experiences. Even if the algorithm is temporarily unable to update or incorporate new data, the system may still execute a valid existing decision rule and continue selecting actions. These issues immediately trigger automated notifications and appear on the monitoring dashboard. However, because they do not directly compromise participant experience or data integrity, the urgency to resolve them is lower, allowing the RL development team more time to address them. The RL algorithm’s ability to learn or update the JITAI decision rules can be compromised in multiple ways, including: (1) in the case of action selection, not obtaining thestate data required by the JITAI decision rules in atimely manner, and (2) in the case of learning, failing to acquire necessary, prior state and reward data from the relevant database.
3. *Green Severity (ImpactsData Analyses Post-deployment).* Green Severity issues represent inconsistencies or events that do not immediately affect the participant nor the RL algorithm but *must* be documented and passed to the data analyst to ensure valid post-deployment analyses. Not documenting these issues compromises the validity of the data analyses and/or hinders improving the JITAI. This category includes two main types of tasks: (i) documenting red and yellow severity issue details and resolutions; and (ii) documenting issues involving components outside of the RL algorithm (e.g., mobile app or backend controller), maintaining a list of items to investigate, and checking the consistency of data saved by all software components. Since managing these issues involves documentation by other components, we recommend establishing agreements between all development teams prior to the deployment of the JITAI software support system. These agreements should clarify responsibilities for documenting specific green severity issues that may arise.

### Fallback methods

Fallback methods are pre-specified procedures executed when an issue occurs, serving as a backup to action selection and learning procedures. These methods ensure that even when red or yellow issues occur, the system defaults to baseline functionality agreed upon before deployment. Specifically, when the RL algorithm’s policy (i.e. JITAI decision rules) fails to output action-selection probabilities or assign actions, individuals should still be assigned an action (e.g. whether to send an intervention prompt or not). If there are any issues with forming or preparing the data for updating the RL algorithm’s policy, then incorrect or corrupted data should be flagged and excluded from the update.

Fallback methods reduce the severity of red issues to yellow. However, it is not feasible to reduce all red issues to yellow because: (1) the appropriate fallback method is not always apparent (e.g., the fallback method is not apparent whendata needed for data analyses does not appear to be recorded in the database) and (2) the ability to identify a red issue may only occur at intervals of time, making it impossible to retroactively address them (e.g., determining whether an excessive number of burdensome interventions have been assigned over a week is only possible at the end of the week).

### Inclusion and ethics statement

This paper *does not* concern human research participant’s data; it concerns the development of software core for monitoring algorithms. For completeness, the following provides further description of the two case studies that motivated this work.

Recruitment for the Oralytics trial was carried out at University of California, Los Angeles (UCLA) dental clinics. The study protocol and consent procedures were approved by the University of California, Los Angeles Institutional Review Board (IRB#21-001471) and the trial was registered on ClinicalTrials.gov (NCT05624489). The trial was conducted from September 2023 to July 2024 with 79 participants. Inclusion criteria were: (a) adults, aged 18 years or older; (2) fluent in English; (3) dentulous or partially edentulous, having at least 16 teeth, with a minimum of 2 teeth in each quadrant; (4) own an iOS or Android smartphone with an active data plan; (5) have a home Wi-Fi system; and (6) willing to consent for passive data collection on tooth-brushing activities in a home setting for 10 weeks. Exclusion criteria were: (1) under 18 years of age; (2) non-English speakers or not fluent in English; (3) lacking any teeth (edentulous) or at least 2 teeth in any quadrant; (4) without a compatible smartphone or a data plan; (5) without access to a home WiFi system; (6) unwilling or unable to consent to passive data collection on tooth-brushing activities for the specified period; and (7) have health conditions or circumstances that might interfere with the study’s data collection, including severe oral or systemic health issues that significantly alter normal tooth-brushing habits, or erratic living situations where consistent data collection is not feasible.

Recruitment for the MiWaves trial, was through U.S. centric social media advertisements. The trial was approved by the University of Michigan Institutional Review Board (IRB#HUM00222194) and the trial was registered on ClinicalTrials.gov (NCT05824754). The trial was conducted from March 2024 to May 2024, with 122 participants. Inclusion criteria were:1) reside in the US; 2) speak and read English; 3) are 18-25 years of age; 4) have a smartphone on which the MiWaves app can be downloaded; 5) screen positive for cannabis use ≥3 times each week over the past month; 6) report any motivation to change cannabis use, operationalized as reporting 1 or greater motivation to reduce or stop cannabis use on a scale ranging from of 0 [Not at all] to 10 [Very]; and 7) meet study verification criteria (i.e., use of CAPTCHA, IP address and social media checks, survey time duration). Exclusion criteria were: 1) participants who report current medical cannabis use and/or fail identity verification procedures.

## Supplementary information

Supplementary Information

## Data Availability

Data sharing is not applicable as this manuscript does not report data generation or analysis.

## Code availability

Both Oralytics and MiWaves RL software systems are open-source and can be found here and here, respectively.
