# Supplementary material for: Effective monitoring of online AI decision-making algorithms in just-in-time adaptive interventions
Source: NPJ Digit Med. 2026 May 4;9:655. doi: 10.1038/s41746-026-02669-4 (PMC13518862; doi:10.1038/s41746-026-02669-4)
Supplement: Supplementary file 1 [file 41746_2026_2669_MOESM1_ESM.pdf]

A Additional Yellow Severity Issues

Here we list and describe all the yellow severity issues that occurred during each respective trial.

A.1 Oralytics

There were three types of yellow severity issues encountered during the Oralytics trial:

1. The RL algorithm was unable to obtain current state (i.e., app analytics data) from the backend controller which affected the data used to update parameters. This happened on two separate occasions for two different participants: (a) the first occasion was because too many requests were made as more participants had joined the trial and the backend controller communicating between the external system and the RL algorithm to provide this data started ignoring the RL algorithm's requests after a certain threshold was exceeded and (b) the second occasion was because the raw participant's data given by the backend controller was malformed and could not be processed. In both cases, fallback method (iii) was executed and the RL algorithm saved the affected data points from those days for the participants affected, but did not add those data points to the batch data used for updating the JITAI decision rules. To solve (a), the RL development team worked with the backend controller team to create a more efficient request strategy that would not exceed the request threshold imposed by the external source and to solve (b), the RL development team worked with the backend controller team to ensure the raw participant's data was no longer malformed.

2. On two occasions, the RL algorithm crashed and was unavailable. However, because of fallback method (i), participants were assigned intervention prompts from the most recent backup intervention schedule saved on their Oralytics app, however, no new schedules were formed for that day. The RL development team restarted the system and the RL algorithm became available again the next day.

3. During action selection by the RL algorithm's JITAI decision rules, the algorithm lost connection to its internal database and was unable to obtain state data from its internal database on three separate occasions. At each occasion, fallback method (ii) was executed and non-personalized schedules were provided for all affected participants. After being notified of each occasion, the RL development team restarted the RL algorithm's internal database and monitored the next week. The exact cause of this issue is still unknown and the RL development team is currently investigating to prevent this issue in the next implementation.

A.2 MiWaves

In MiWaves, the majority of the yellow severity issues were aimed at verifying the functionality of the RL algorithm and it's components, and checking for any associated issues. Three broad instances of yellow severity issues encountered during the MiWaves clinical trial are specified below:

1. The RL algorithm utilized consistent and accurate participant data for updating the JITAI decision rules. However, the RL algorithm encountered a problem prior to an update where it received duplicate or empty responses for participant data from the backend controller after the end of a given decision point. The development teams discovered that when a participant traveled to a different time zone and missed their self-monitoring period, the backend controller would skip the scheduled tasks for that decision point. As a result, no data was available for the RL algorithm. Since this issue occurred infrequently (in less than 3% of decision points), the teams decided not to fix it immediately during the trial. However, the RL algorithm excluded the data associated with these decision points to update the algorithm (this was the fallback method).

2. If the RL algorithm received an empty response concerning a particular participant from the backend controller (as seen in scenario 1), the RL algorithm could not update the current time of day for that participant. This issue also affected all subsequent time-of-day values (necessary for the RL algorithm's state) for that participant. The app and RL development team quickly implemented a solution within two business days to fix the issue. They added a label to specify the time of day when the backend controller communicated with the RL algorithm, which the RL algorithm would then use to correctly determine the time for each participant in the trial.

3. On the first day of the MiWaves trial, certain participants were removed after the trial team discovered that they were fraudulent (see Appendix I). However, this information was not communicated to the RL algorithm. As a result, there was a discrepancy between the actual number of active participants in the trial and the number perceived to be active by the RL algorithm. This mismatch caused the RL algorithm's decision rules to incorrectly assign actions to the participants (participant A was incorrectly assigned the action for participant B). Within one day, the development teams addressed the issue by implementing a fix (in both the backend controller and the RL algorithm) and restarting the RL algorithm to prevent similar scenarios in the future. The fix involved adding a flag to communicate about active participants, and henceforth the RL algorithm maintaining the correct set of active participants in the trial.

B Oralytics RL Algorithm

For completeness and to clarify ideas in the main paper, we offer an overview of key components of the Oralytics RL algorithm. See the Oralytics RL design manuscripts [1, 2] for full details on the Oralytics RL algorithm.

B.1 Definitions

We use $i \in [1 : N]$ to denote participants and $t \in [1 : T]$ to denote decision points. $S_{i,t} \in \mathbb{R}^d$ denotes the vector of covariate features of dimension d (num. of features) used for participant i's current state at decision point t. $A_{i,t}$ denotes the action (i.e., intervention option) selected for participant i's current state at decision point t. Recall that in our case, action selection means selecting between sending an engagement prompt or not. $Q_{i,t}$ denotes the proximal health outcome of oral self-care behaviors (OSCB) measured in seconds, observed after action $A_{i,t}$ is executed.

B.2 Reinforcement Learning Framework

- **state**: Let $S_{i,t} \in \mathbb{R}^d$ represent the vector of covariate features of dimension $d = 5$ (num. of features) used as participant i's current state at decision point t by the RL algorithm:

    1. Time of Day (Morning/Evening) $\in \{0, 1\}$
    2. $\bar{B}$: Exponential Average of Brushing Quality Over Past 7 Days (Normalized) $\in [-1, 1]$
    3. $\bar{A}$: Exponential Average of Prompts Sent Over Past 7 Days (Normalized) $\in [-1, 1]$
    4. Prior Day App Engagement (Opened App / Not Opened App) $\in \{0, 1\}$
    5. Intercept Term $= 1$

  Feature 1 is 0 for morning and 1 for evening. Features 2 and 3 are $\bar{B}_{i,t} = c_\gamma \sum_{j=1}^{14} \gamma^{j-1} Q_{i,t-j}$ and $\bar{A}_{i,t} = c_\gamma \sum_{j=1}^{14} \gamma^{j-1} A_{i,t-j}$ respectively, where $\gamma = 13/14$ and $c_\gamma = \frac{1-\gamma}{1-\gamma^{14}}$. Recall that $Q_{i,t}$ is the proximal outcome of OSCB and $A_{i,t}$ is the action. Feature 4 is 1 if the participant has opened the app in focus (i.e., not in the background) the prior day and 0 otherwise. Feature 5 is the intercept which is always 1. Please refer to Section 2.7 in [1] for full details on the design of the state.

- **Actions**: Binary actions, i.e. $\mathcal{A} = \{0, 1\}$ - to not send an intervention message (0) or to send an intervention message (1).

- **Decision Points**: $T = 140$ decision points per participant. For each participant, the Oralytics clinical trial ran for 70 days, and each day had 2 decision points (i.e., morning and evening).

- **Reward**: Let $R_{i,t} \in \mathbb{R}$ denote the reward for participant i after taking action $A_{i,t}$ at decision point t. We defined the reward $R_{i,t}$ given to the algorithm to be a function of brushing quality $Q_{i,t}$ (i.e., proximal health outcome) in order to improve the algorithm's learning [3]. The reward $R_{i,t}$ for the ith participant at decision point t is:

$$R_{i,t} := Q_{i,t} - C_{i,t} \tag{1}$$

The cost term $C_{i,t}$ allows the RL algorithm to optimize for immediate healthy brushing behavior, while also considering the delayed effects of the current action on the effectiveness of future actions. The cost term is a function (with parameters ξ_1, ξ_2) which takes in current state $S_{i,t}$ and action $A_{i,t}$ and outputs the delayed negative effect of currently sending a prompt.

Let $\bar{B}_{i,t}$ and $\bar{A}_{i,t}$ be state features 2 and 3 defined earlier. The cost of sending a prompt is:

$$C_{i,t} := \begin{cases} \xi_1 \mathbb{I}[\bar{B}_{i,t} > b]\mathbb{I}[\bar{A}_{i,t} > a_1] \\ \qquad + \xi_2 \mathbb{I}[\bar{A}_{i,t} > a_2] & \text{if } A_{i,t} = 1 \\ 0 & \text{if } A_{i,t} = 0 \end{cases} \tag{2}$$

B.3 Action Selection and Policy Update

The algorithm's policy (JITAI decision rule) selects actions by deciding between sending a prompt $A_{i,t} = 1$ or not $A_{i,t} = 0$. If $A_{i,t} = 1$, the backend controller (described in Section 2.1) randomly selected content for each prompt. There are 3 different content categories: (1) participant winning a gift (direct reciprocity), (2) participant winning a gift for their favorite charity (reciprocity by proxy), and (3) Q&A for the morning decision point or feedback on prior brushing for the evening decision point. To decide on the exact content, the main controller first samples a category with equal probability and then samples with replacement prompt content from that category. Due to the large number of prompt content in each category, it is highly unlikely that a participant received the same content twice.

We now discuss how the algorithm learns and selects actions using its policy (JITAI decision rules). Since the RL algorithm in Oralytics is a generalized contextual bandit, the model of the participant environment is a model of the conditional mean reward (reward given current state and selected action). To model the conditional mean reward, the Oralytics RL algorithm uses a Bayesian Linear Regression with action centering model:

$$R_{i,t} = S_{i,t}^T \alpha_0 + \pi_{i,t} S_{i,t}^T \alpha_1 + (A_{i,t} - \pi_{i,t}) S_{i,t}^T \beta + \epsilon_{i,t} \tag{3}$$

where $S_{i,t}$ is the algorithm's state (defined in Appendix B.2), $\pi_{i,t}$ is the probability that the RL algorithm's

policy selects action $A_{i,t} = 1$ in state $S_{i,t}$. $\epsilon_{i,t} \sim \mathcal{N}(0, \sigma^2)$ and there are priors on $\alpha_0 \sim \mathcal{N}(\mu_{\alpha_0}, \Sigma_{\alpha_0})$, $\alpha_1 \sim \mathcal{N}(\mu_\beta, \Sigma_\beta)$, $\beta \sim \mathcal{N}(\mu_\beta \Sigma_\beta)$.

Policy Update To learn throughout the trial, the algorithm updates its policy parameters. Let $\tau(i,t)$ be the update time for participant i that included the current decision point t. We needed an additional index because update times τ and decision points t are not on the same cadence. At policy update time, the reward model's posterior parameters are updated with all history of state, actions, and rewards up to that point. Since the RL algorithm uses posterior sampling to determine action-selection probabilities, the algorithm updates parameters $\mu^{\text{post}}_{\tau(i,t)}$ and $\Sigma^{\text{post}}_{\tau(i,t)}$ of the posterior distribution.

Action Selection The RL algorithm for Oralytics is a modified posterior sampling algorithm called the smooth posterior sampling algorithm. The algorithm's policy selects action $A_{i,t} \sim \text{Bern}(\pi_{i,t})$:

$$\pi_{i,t} = \mathbb{E}_{\tilde{\beta} \sim \mathcal{N}(\mu^{\text{post}}_{\tau(i,t)-1}, \Sigma^{\text{post}}_{\tau(i,t)-1})} \left[\rho(s^\top \tilde{\beta}) \middle| \mathcal{H}_{1:n,\tau(i,t)-1}, S_{i,t} = s \right] \tag{4}$$

$\tau(i,t) - 1$ is the last update time for the posterior parameters. Notice that the last expectation above is only over the draw of $\tilde{\beta}$ from the posterior distribution.

In smooth posterior sampling, ρ is a smooth function chosen to enhance the replicability of the randomization probabilities if the study is repeated [4]. The Oralytics RL algorithm set ρ to be a generalized logistic function:

$$\rho(x) = L_{\min} + \frac{L_{\max} - L_{\min}}{\left[1 + c \exp(-bx)\right]^k} \tag{5}$$

where asymptotes $L_{\min} = 0.2, L_{\max} = 0.8$ are clipping values (bounded away from 0 and 1) to enhance the ability to answer scientific questions with sufficient power [5]. This way $\pi_{i,t}$ can only attain values within $[L_{\min} = 0.2, L_{\max} = 0.8]$.

B.4 Backup Intervention Schedule

A critical design decision made for Oralytics was to construct a backup intervention schedule (for the full 70 days duration of the intervention) every morning rather than only selecting actions for the current day. For Oralytics, the backend controller and mobile app were designed and developed separately by different teams. Therefore, there's a higher likelihood of integration and communication issues that could arise that impact intervention delivery. This decision mitigates possible communication issues between the backend controller and mobile app which could lead to participants failing to obtain actions for the current decision point.

Recall that after the update of the model parameters, the RL algorithm constructs a policy (i.e., JITAI decision rule) based on the updated parameters. The algorithm uses the policy, combined with each participant's state to select actions at that decision point. For decision points of the current day, the RL algorithm has current state. However the state is unknown for future decision points. Therefore, for future decision points we use either a modified state or do not use any state and select actions with fixed probability. More specifically, the backup intervention schedule is constructed as follows:

- For the current day's decision points, $j = t, t+1$, the algorithm uses the current state $S_{i,t}, S_{i,t+1}$ and the current policy to select actions.
- For decision points within the first 2 weeks from t, $j = t+2, t+3, ..., t+26, t+27$, the algorithm uses a modified state (see Appendix B.4.1) and the current policy to select actions.
- For all decision points after $t+27$, the algorithm select actions with a fixed probability 0.5.

B.4.1 Modified Feature Space for Fallback Method

For decision points within the first 2 weeks from the current day, the algorithm uses the state specified in Appendix B.2 with the following modifications:

- Feature 2 is replaced with the *Most Recent* Exponential Average Brushing Quality Over Past 7 Days (Normalized)
- Feature 4 is replaced with the *Best Guess of* Prior Day App Engagement (Opened App / Not Opened App) = 0

Of the state features presented earlier, features 5, 1, and 3 are known except for features 2 ($\bar{B}$) and 4 (prior day app engagement). Since future values of $\bar{B}$ are unknown, we impute this feature with the most recent value of $\bar{B}_{i,t}$ known when the schedule was formed. Namely, the $\bar{B}$ value for decision points $j = t+2, ..., t+27$ used the same $\bar{B}$ value as decision points $t, t+1$. For the prior day app engagement feature, we impute the value to 0, because our best guess is that the participant does not getting a fresh schedule because they did not open the app.

C MiWaves RL Algorithm

For completeness and to clarify ideas in the main paper, we also offer an overview of key components of the MiWaves RL algorithm. Please refer to the MiWaves RL algorithm design manuscripts[6, 7] for full details

on the MiWaves RL algorithm. We use $i \in [1:m]$ to denote participants and $t \in [1:T]$ to denote decision points.

C.1 Reinforcement Learning Framework

This section provides a brief overview of the Reinforcement Learning (RL) framework used in this work with respect to the MiWaves trial.

- **state**: Let $\boldsymbol{S_i^{(t)}} \in \mathbb{Z}_2^d$ represent the vector of binary covariates of dimension $d = 3$ (number of features) used as participant i's current state at a given decision point t by the RL algorithm. It is defined as a 3 dimensional tuple of binary features, i.e. $\boldsymbol{S_i^{(t)}} = \{S_{i1}^{(t)}, S_{i2}^{(t)}, S_{i3}^{(t)}\}$ where:
  - $S_{i1}^{(t)}$: Recent app engagement (Low / High) over the past 3 days $\in \{0, 1\}$
  - $S_{i2}^{(t)}$: Time of day (Morning / Evening) $\in \{0, 1\}$
  - $S_{i3}^{(t)}$: Recent cannabis use reported during the most recent self-monitoring period (Using / Not using) $\in \{0, 1\}$

  $S_{i2}^{(t)}$ is 0 for morning and 1 for evening. For $S_{i1}^{(t)}$ and $S_{i3}^{(t)}$, the less favorable states are represented as 0 (using cannabis, low app engagement), and the more favorable ones are represented as 1 (not using cannabis, high app engagement). Please refer to Section 1.3 in the MiWaves RL design manuscript[8] for full details on the design of the state space.

- **Actions**: Binary actions, i.e. $\mathcal{A} = \{0, 1\}$ - to not send an engagement prompt (0) or to send an engagement prompt (1).

- **Decision Points**: $T = 60$ decision points per participant. For each participant, the MiWaves clinical trial ran for 30 days, and each day had 2 decision points per day. Therefore, we had 60 decision points per participant.

- **Reward**: Let us denote the reward for participant i after taking an action at decision point t by $R_i^{(t+1)} \in \{0, 1, 2, 3\}$. One of the assumptions underpinning the MiWaves clinical trial is that higher app and intervention engagement (self-monitoring activities and using suggestions by MiWaves to manage mood, identify alternative activities, plan goals, etc.) will lead to lower cannabis use. Hence, the higher levels of reward correspond to higher levels of engagement by the participant, and vice-versa.

C.2 Action Selection and Parameter Updating

We now discuss how the algorithm learns and selects actions. Since the RL algorithm in MiWaves is a generalized contextual bandit, the model of the participant environment is a model of the conditional mean reward (reward given current state and selected action). To model the conditional mean reward, the MiWaves RL algorithm utilizes a Bayesian Linear Mixed Model with action centering [9]. For a given participant i at decision point t, the RL algorithm receives the reward $R_i^{(t+1)}$ after taking action $a_i^{(t)}$ in the participant's current state $\boldsymbol{S_i^{(t)}}$. Then, the algorithm's reward approximation model for participant i is written as:

$$R_i^{(t+1)} = g(\boldsymbol{S_i^{(t)}})^T \boldsymbol{\alpha_i} + (a_i^{(t)} - \pi_i^{(t)}) f(\boldsymbol{S_i^{(t)}})^T \boldsymbol{\beta_i} + (\pi_i^{(t)}) f(\boldsymbol{S_i^{(t)}})^T \boldsymbol{\gamma_i} + \epsilon_i^{(t)} \tag{6}$$

where $\epsilon_i^{(t)}$ is the noise, assumed to be gaussian i.e. $\boldsymbol{\epsilon} \sim \mathcal{N}(\mathbf{0}, \sigma_\epsilon^2 \boldsymbol{I}_{tm_t})$, and m_t is the total number of participants who have been or are currently part of the MiWaves digital intervention at time t. Also $\boldsymbol{\alpha_i}$, $\boldsymbol{\beta_i}$, and $\boldsymbol{\gamma_i}$ are weights that the algorithm wants to learn. $\pi_i^{(t)}$ is the probability of taking action $a_i^{(t)} = 1$ in state $\boldsymbol{S_i^{(t)}}$ for participant i at decision point t. $g(\boldsymbol{S_i^{(t)}})$ and $f(\boldsymbol{S_i^{(t)}})$ are functions of the algorithm's state (defined in Appendix C.1), defined as:

$$g(\boldsymbol{S_i^{(t)}}) = f(\boldsymbol{S_i^{(t)}}) = [1, S_{i1}^{(t)}, S_{i2}^{(t)}, S_{i3}^{(t)}, S_{i1}^{(t)} S_{i2}^{(t)}, S_{i2}^{(t)} S_{i3}^{(t)}, S_{i1}^{(t)} S_{i3}^{(t)}, S_{i1}^{(t)} S_{i2}^{(t)} S_{i3}^{(t)}] \tag{7}$$

We refer to the term $g(\boldsymbol{S_i^{(t)}})^T \boldsymbol{\alpha_i}$ as the baseline, and $f(\boldsymbol{S_i^{(t)}})^T \boldsymbol{\beta_i}$ as the advantage (i.e. the advantage of taking action 1 over action 0).

We re-write the reward model as follows:

$$R_i^{(t+1)} = \boldsymbol{\Phi_{it}^T} \boldsymbol{\theta_i} + \epsilon_i^{(t)} \tag{8}$$

where $\boldsymbol{\Phi_{it}^T} = \boldsymbol{\Phi}(\boldsymbol{S_i^{(t)}}, a_i^{(t)}, \pi_i^{(t)})^T = [g(\boldsymbol{S_i^{(t)}})^T, (a_i^{(t)} - \pi_i^{(t)}) f(\boldsymbol{S_i^{(t)}})^T, (\pi_i^{(t)}) f(\boldsymbol{S_i^{(t)}})^T]$ is the design matrix for given state and action, and $\boldsymbol{\theta_i} = [\boldsymbol{\alpha_i}, \boldsymbol{\beta_i}, \boldsymbol{\gamma_i}]^T$ is the joint weight vector that the algorithm wants to learn. We further break down the joint weight vector $\boldsymbol{\theta_i}$ into two components:

$$\boldsymbol{\theta_i} = \begin{bmatrix} \boldsymbol{\alpha}_i \\ \boldsymbol{\beta}_i \\ \boldsymbol{\gamma}_i \end{bmatrix} = \begin{bmatrix} \boldsymbol{\alpha}_{\text{pop}} + \boldsymbol{u}_{\alpha,i} \\ \boldsymbol{\beta}_{\text{pop}} + \boldsymbol{u}_{\beta,i} \\ \boldsymbol{\gamma}_{\text{pop}} + \boldsymbol{u}_{\gamma,i} \end{bmatrix} = \boldsymbol{\theta}_{\text{pop}} + \boldsymbol{u_i} \tag{9}$$

Here, $\boldsymbol{\theta}_{\text{pop}} = [\boldsymbol{\alpha}_{\text{pop}}, \boldsymbol{\beta}_{\text{pop}}, \boldsymbol{\gamma}_{\text{pop}}]^T$ is the population level term which is common across all the participant's reward models. We assume a gaussian prior distribution on $\boldsymbol{\theta}_{\text{pop}}$ given by $\boldsymbol{\theta}_{\text{pop}} \sim \mathcal{N}(\boldsymbol{\mu}_{\text{prior}}, \boldsymbol{\Sigma}_{\text{prior}})$. On

the other hand, $\boldsymbol{u_i} = [\boldsymbol{u}_{\alpha,i}, \boldsymbol{u}_{\beta,i}, \boldsymbol{u}_{\gamma,i}]^T$ are the individual level parameters, or the *random effects*, for any given participant i. Note that the individual level parameters are assumed to be gaussian by definition, i.e. $\boldsymbol{u_i} \sim \mathcal{N}(\mathbf{0}, \boldsymbol{\Sigma_u})$.

Parameter Updating To learn, the algorithm updates its parameters daily (after every even numbered decision point t). At parameter update time, the reward model's posterior parameters are updated with all history of state, actions, and rewards up to that point. The MiWaves RL algorithm uses a variant of Thompson sampling, and hence the algorithm updates parameters $\boldsymbol{\mu}^{(\boldsymbol{t})}_{\text{post}}$ and $\boldsymbol{\Sigma}^{(\boldsymbol{t})}_{\text{post}}$ of the posterior distribution. The updated posteriors are given as:

$$\boldsymbol{\mu}^{(\boldsymbol{t})}_{\text{post}} = \left(\tilde{\boldsymbol{\Sigma}}^{-1}_{\boldsymbol{\theta,t}} + \frac{1}{\sigma^2_\epsilon}\boldsymbol{A}\right)^{-1}\left(\tilde{\boldsymbol{\Sigma}}^{-1}_{\boldsymbol{\theta,t}}\boldsymbol{\mu_\theta} + \frac{1}{\sigma^2_\epsilon}\boldsymbol{B}\right) \tag{10}$$

$$\boldsymbol{\Sigma}^{(\boldsymbol{t})}_{\text{post}} = \left(\tilde{\boldsymbol{\Sigma}}^{-1}_{\boldsymbol{\theta,t}} + \frac{1}{\sigma^2_\epsilon}\boldsymbol{A}\right)^{-1} \tag{11}$$

where

$$\boldsymbol{A} = \begin{bmatrix} \sum_{\tau=1}^t \boldsymbol{\Phi_{1\tau}}\boldsymbol{\Phi_{1\tau}^T} & \mathbf{0} & \cdots & \mathbf{0} \\ \mathbf{0} & \sum_{\tau=1}^t \boldsymbol{\Phi_{2\tau}}\boldsymbol{\Phi_{2\tau}^T} & \cdots & \mathbf{0} \\ \vdots & \vdots & \ddots & \vdots \\ \mathbf{0} & \mathbf{0} & \cdots & \sum_{\tau=1}^t \boldsymbol{\Phi_{m_t\tau}}\boldsymbol{\Phi_{m_t\tau}^T} \end{bmatrix} \tag{12}$$

$$\boldsymbol{B} = \begin{bmatrix} \sum_{\tau=1}^t \boldsymbol{\Phi_{1\tau}} R_1^{(\tau+1)} \\ \vdots \\ \sum_{\tau=1}^t \boldsymbol{\Phi_{m_t\tau}} R_{m_t}^{(\tau+1)} \end{bmatrix} \quad \boldsymbol{\mu_\theta} = \begin{bmatrix} \boldsymbol{\mu}_{\text{prior}} \\ \boldsymbol{\mu}_{\text{prior}} \\ \vdots \\ \boldsymbol{\mu}_{\text{prior}} \end{bmatrix} \tag{13}$$

$$\tilde{\boldsymbol{\Sigma}}_{\boldsymbol{\theta,t}} = \begin{bmatrix} \boldsymbol{\Sigma}_{\text{prior}} + \boldsymbol{\Sigma_u} & \boldsymbol{\Sigma}_{\text{prior}} & \cdots & \boldsymbol{\Sigma}_{\text{prior}} \\ \boldsymbol{\Sigma}_{\text{prior}} & \boldsymbol{\Sigma}_{\text{prior}} + \boldsymbol{\Sigma_u} & \cdots & \boldsymbol{\Sigma}_{\text{prior}} \\ \vdots & \vdots & \ddots & \vdots \\ \boldsymbol{\Sigma}_{\text{prior}} & \boldsymbol{\Sigma}_{\text{prior}} & \cdots & \boldsymbol{\Sigma}_{\text{prior}} + \boldsymbol{\Sigma_u} \end{bmatrix} \tag{14}$$

The estimates of σ^2_ϵ and $\boldsymbol{\Sigma_u}$ are updated at the end of each week. The update procedure for these estimates can be found in Section 2.2.2 of the MiWaves RL design manuscript [8, 7].

Action Selection The MiWaves RL algorithm utilizes a variant of posterior sampling called the smooth posterior sampling for action selection. The action selection procedure utilizes the Gaussian posterior distribution defined by the posterior mean $\boldsymbol{\mu}_{\text{post}}^{\boldsymbol{(t-1)}}$ and variance $\boldsymbol{\Sigma}_{\text{post}}^{\boldsymbol{(t-1)}}$ to determine the action selection probability $\pi^{(t)}$ and the corresponding action $a^{(t)}$ for the given time step t as follows:

$$\pi_i^{(t)} = \mathbb{E}_{\tilde{\beta}\sim\mathcal{N}(\mu_{\text{post},i}^{(t-1)},\Sigma_{\text{post},i}^{(t-1)})}[\rho(f(\boldsymbol{S_i^{(t)}})^T\tilde{\boldsymbol{\beta}})|\mathcal{H}_{1:m_{t-1}}^{(t-1)}, \boldsymbol{S_i^{(t)}}] \tag{15}$$

where $\mathcal{H}_{1:m_{t-1}}^{(t-1)} = \{\boldsymbol{S_i^{(1)}}, a_i^{(1)}, R_i^{(2)}, \cdots, \boldsymbol{S_i^{(t-1)}}, a_i^{(t-1)}, R_i^{(t)}\}_{i\in[m_{t-1}]}$ refers to the trajectories (history of state action reward tuples) from time $\tau = 1$ to $\tau = t-1$ for all participants $i \in [m_{t-1}]$. Notice that the last expectation above is only over the draw of β from the posterior distribution parameterized by $\boldsymbol{\mu}_{\text{post},\boldsymbol{i}}^{\boldsymbol{(t-1)}}$ and $\boldsymbol{\Sigma}_{\text{post},\boldsymbol{i}}^{\boldsymbol{(t-1)}}$.

In smooth posterior sampling, ρ is a smooth function chosen to enhance the replicability of the randomization probabilities if the trial is repeated [4]. The MiWaves RL algorithm set ρ to be a generalized logistic function:

$$\rho(x) = L_{\min} + \frac{L_{\max} - L_{\min}}{\left[1 + c\exp(-bx)\right]^k} \tag{16}$$

where asymptotes $L_{\min} = 0.2, L_{\max} = 0.8$ are clipping values (bounded away from 0 and 1) to enhance the ability to answer scientific questions with sufficient power [5]. This way $\pi_i^{(t)}$ can only attain values within $[L_{\min} = 0.2, L_{\max} = 0.8]$. The algorithm selects actions by deciding between sending a prompt $a_i^{(t)} = 1$ or not $a_i^{(t)} = 0$ as follows:

$$a_i^{(t)} \sim \text{Bern}(\pi_i^{(t)}) \tag{17}$$

If $a_i^{(t)} = 1$, the backend controller (described in Section 2.1) randomly selects the intervention content for each prompt. In the MiWaves clinical trial, there were 6 different content categories depending on the prompt length and the requested interaction level of the participant. More details on the content categories can be found in Supplementary Table 2 of the MiWaves protocol[6].

D Miscellaneous operational and implementation details

D.1 Oralytics

The Oralytics RL development team consisted of one experienced RL algorithm developer and one junior software engineer. The design and implementation of the software infrastructure supporting the RL algorithm spanned approximately one year and involved multiple iterations. However, within the broader development timeline, the monitoring system itself was designed and implemented under 2 months to meet the trial start date. Prior to the main trial, the RL system (including the monitoring framework) was evaluated in two stages: a beta phase conducted with internal staff members and a pilot phase involving nine participants over 35 days.

D.2 MiWaves

The MiWaves RL development team consisted of one experienced researcher with expertise in both reinforcement learning and software engineering. The system architecture, including the comprehensive monitoring framework, was designed over a three-week planning phase followed by a three-month implementation period. To ensure system robustness, the RL algorithm system underwent rigorous evaluation across two beta testing phases involving 5–10 participants each prior to clinical deployment.

E Potential Issues for Oralytics

Severity	EC	Description
RED	R1	Algorithm has assigned no intervention prompts to participant {`participant id`} for every decision point in the last 7 days
	R2	Algorithm has assigned intervention prompts to participant {`participant id`} for every decision point in the last 7 days
	R3	Error in saving data for participant {`participant id`}.
YELLOW	Y1	Dependency endpoint call to {`endpoint name`} failed for participant {`participant id`}.
	Y2	Endpoint response from {`endpoint name`} cannot be json-ified for participant {`participant id`}.
	Y3	Endpoint call to {`endpoint name`} returned malformed data for participant {`participant id`}.

	Y4	Endpoint call to {`endpoint name`} returned no data for participant {`participant id`}.
	Y5	Action selection procedure failed and fallback method was executed for participant {`participant id`}.
	Y6	Algorithm could not update policy (posterior parameters) with given batch data.
	Y7	RL Service and endpoints are down. No new schedules formed.

Supplementary Table 1: Oralytics monitoring issue severities. {`endpoint name`} refers to the type of dependency endpoint that was called and {`participant id`} refers to unique identifier assigned to the participant for the trial

F Potential Issues for MiWaves

Severity	EC	Description
RED	R1	For a given decision point, more than 80% of the active participants (started and did not complete 30 days yet in the trial) did not receive an intervention message.
	R2	For a given decision point, more than 80% of the active participants (started and did not complete 30 days yet in the trial) received an intervention message.
	R3	For any given participant at any time, in the last 7 days, they received (i.e. get assigned) intervention messages for more than 80% of the decision points.
	R4	For any given participant at any time, in the last 7 days, they did not receive (i.e. get assigned) any intervention messages for more than 80% of the decision points.
	R5	The action selection probability returned by the RL algorithm for any participant is greater than 0.8 or less than 0.2
YELLOW	0	Authorization token is malformed.
	1	Authorization token is missing
	2	Client's authorization token is invalid/does not match
	3	Something went wrong with the authorization procedure on the RL API
	4	No more client/backends allowed to register on RL API
	5	Error occurred while trying to register client/backend on RL API
	6	Client/Backend already exists on RL API database
	7	Invalid client/backend auth credentials
	8	Something went wrong trying to authenticate client/backend credentials

9	Failure in trying to log out client - as RL API was not able to blacklist auth token and commit to db
10	Malformed request while trying to log out client
11	Invalid auth token to log out
100	participant id was invalid
101	RL start date is invalid
102	RL end date is invalid
103	Consent start date is invalid
104	Consent end date is invalid
105	Morning notification time is invalid
106	Evening notification time is invalid
107	Some error occurred trying to push the participant registration info to RL database
108	Participant has already been registered
109	Some error occurred during participant registration procedure
200	Participant id was invalid
201	Finished ema flag is invalid
202	App use flag is invalid
203	Participant id does not exist in the RL database
204	Error trying to construct reward by the RL algorithm
205	Error trying to construct state by the RL algorithm
206	Error trying to compute action by the RL algorithm
207	Some unknown error occurred trying to compute action for given participant
208	Some unknown error occurred trying to compute action for given participant
209	Activity question response field is not present when ema has been completed
210	Cannabis use field is not present or empty when ema has been completed
211	Window label field is not present or empty
300	Participant ID does not exist
301	The trial has not started for the given participant
302	The trial has ended for the given participant
303	Some unknown error occurred trying to commit participant data and end the participant decision window
304	Invalid action taken passed by caller

	305	Invalid action selection seed passed by caller
	306	Invalid probability of action taken passed by caller
	307	Invalid policy id passed by caller
	308	Invalid decision index passed by caller
	309	Invalid action generation timestamp passed by caller
	310	Invalid action request ID (rid) passed by caller
	311	Invalid EMA finished timestamp passed by caller
	312	Invalid push notification timestamp passed by caller
	313	Invalid message click notification timestamp passed by caller
	314	Invalid morning notification time passed by caller
	315	Invalid evening notification time passed by caller
	316	Query to backend to fetch participant data failed
	317	Empty data returned by backend when trying to fetch data for given participant for the ending decision window
	318	More than one decision window's data returned by the backend
	319	No action found in RL database for the given action request ID (rid provided by caller)
	320	Some unknown error occurred trying to fetch data and update the decision point for given participant
	321	Some error occurred while trying to update the algorithm's data records for this decision point index
	322	Decision index already exists in the database (duplicate entry returned by the backend)
	323	Some unknown error occurred trying to check if the decision index does not exist in the database
	324	Invalid window label
	400	RL API failed to dump database tables to disk
	401	RL API failed to add new RL weights to database
	402	Some unknown error occurred during RL algorithm update
	403	RL failed to update hyper-parameters
	404	RL failed to update posteriors
	405	RL update's use data flag is invoked but not implemented
	406	Error while constructing return parameters after RL update

Supplementary Table 2: MiWaves monitoring issue severities. EC refers to the Error Code which was used in the automated email to report for errors.

G Oralytics Database Schema

Oralytics maintained 5 data tables:

1. **Participant Info Table:** Keeps track of trial participants and participant information
2. **Posterior Weights Table:** Stores the posterior mean and variance of the RL algorithm
3. **Participant Data Table:** Stores every data row from every schedule formed for that participant
4. **Action Selection Data Table:** Stores the participant data row corresponding to the action that was actually executed, including state (may or may not be imputed) used by the algorithm and the components of the reward for that participant decision point
5. **Update Data Table:** Stores data rows corresponding to state, action, reward, and the action selection probability to update the RL algorithm

G.1 Data Tables and Values

G.1.1 Participant Info Table

- `participant_id`: unique participant identifier
- `participant_start_day`: first day that the participant enters the trial
- `participant_end_day`: last day that the participant is in the trial
- `morning_time_weekday`: participant-specific weekday morning decision point
- `evening_time_weekday`: participant-specific weekday evening decision point
- `morning_time_weekend`: participant-specific weekend morning decision point
- `evening_time_weekend`: participant-specific weekend evening decision point
- `participant_entry_decision_t`: trial-level decision point when the participant enters the trial
- `participant_last_decision_t`: trial-level decision point when the participant exits the trial

- `currently_in_trial`: 1 if the participant is currently in the trial, 0 otherwise
- `participant_day_in_trial`: participant-level day in trial [1, 70]
- `participant_opened_app`: 1 if the participant opened the app the prior day, 0 otherwise
- `most_recent_schedule_id`: the schedule id of the schedule of actions most recently obtained by the app

G.1.2 Posterior Weights Table

- `policy_idx`: index for the update time and the policy, starts with 0. 0 index refers to the prior distribution before any data update and 1 index refers to the first posterior update using data.
- `timestamp`: timestamp of policy update
- `posterior_mu.{}`: flattened posterior mean vector where {} indexes into the vector, starts with 0
- `posterior_var.{}.{}`: flattened posterior covariance matrix where {} indexes the row and the second {} indexes the column, starts with 0

G.1.3 Participant Data Table

- `participant_id`: unique participant identifier
- `participant_start_day`: first day that the participant enters the trial
- `participant_end_day`: last day that the participant is in the trial
- `timestamp`: timestamp of when the action from the corresponding schedule_id was created
- `schedule_id`: id of the schedule that this action came from
- `participant_decision_t`: indexes the participant-specific decision point starts with 0, ends with 139 (note: even values denote the morning decision point, odd values denote the evening decision point)
- `decision_time`: unique datetime (i.e., %Y-%m-%d %H:%M:%S) for when the action was executed
- `day_in_trial`: trial-level day in trial
- `policy_idx`: policy used to select action
- `random_seed`: random integer in [0, 999] to reproduce the Bernoulli draw of the action given action-selection probability

- `action int`: {0, 1} where 1 denotes a message being sent and 0 denotes a message not being sent
- `prob`: action selection probability
- `state.{}`: flattened state (state) vector observed by the algorithm at decision point (Note: this is the state used for action-selection as b_bar, a_bar are imputed value depending on what schedule the participant gets.)

G.1.4 Action Selection Data Table

- `participant_id`: unique participant identifier
- `participant_start_day`: first day that the participant enters the trial
- `participant_end_day`: last day that the participant is in the trial
- `timestamp`: timestamp of when the action from the corresponding schedule_id was created
- `schedule_id`: id of the schedule that this action came from
- `participant_decision_t`: indexes the participant-specific decision point starts with 0, ends with 139 (note: even values denote the morning decision point, odd values denote the evening decision point)
- `decision_time`: unique datetime (i.e., %Y-%m-%d %H:%M:%S) for when the action was executed
- `day_in_trial`: trial-level day in trial
- `policy_idx`: policy used to select action
- `random_seed`: random integer in [0, 999] to reproduce the Bernoulli draw of the action given action-selection probability
- `action int`: {0, 1} where 1 denotes a message being sent and 0 denotes a message not being sent
- `prob`: action selection probability
- `state.{}`: flattened state vector observed by the algorithm at decision point (Note: this is the state used for action-selection as b_bar is an imputed value depending on what schedule the participant gets.)
- `brushing_duration`: brushing durations in seconds
- `pressure_duration`: pressure durations in seconds

- `quality`: brushing quality truncated at 180 seconds
- `raw_quality`: actual brushing quality for that decision point
- `reward`: our designed surrogate reward given to the algorithm
- `cost_term`: cost term of surrogate reward
- `B_condition`: B_condition of the cost term
- `A1_condition`: A1_condition of the cost term
- `A2_condition`: A2_condition of the cost term
- `actual_b_bar`: actual observed b_bar state value used in calculating B_condition (notice that a_bar used in calculating A1_condition and A2_condition is the same a_bar in the state value above)

G.1.5 Update Data Table

- `participant_id`: unique participant identifier
- `participant_start_day`: first day that the participant enters the trial
- `participant_end_day`: last day that the participant is in the trial
- `timestamp`: timestamp of when this data tuple was added to the table
- `participant_decision_t`: indexes the participant-specific decision point starts with 0, ends with 139 (note: even values denote the morning decision point, odd values denote the evening decision point)
- `decision_time`: unique datetime (i.e., %Y-%m-%d %H:%M:%S) for when the action was executed
- `first_policy_idx`: first policy idx to use this data tuple for update
- `action int`: {0, 1} where 1 denotes a message being sent and 0 denotes a message not being sent
- `prob`: action selection probability
- `reward`: our designed surrogate reward given to the algorithm
- `quality`: brushing quality truncated at 180 seconds
- `state.{}`: flattened state vector observed by the algorithm at decision point (Note: this is the state used to update the algorithm, b_bar and a_bar are the actual observed values for that decision point)

H MiWaves Database Schema

MiWaves maintained 7 data tables:

1. **Participants Table**: Contains participant-specific data such as unique identifiers, consent periods, and reinforcement learning periods (Supplementary Table 3).
2. **Participant Status Table**: Tracks the status of each participant within the trial, including trial phase, notification times, decision indices, and current trial day (Supplementary Table 4).
3. **Algorithm Status Table**: Stores the status of the algorithm, including policy identifiers, update timestamps, and trial day information (Supplementary Table 5).
4. **RL Hyperparameter Update Request Table**: Logs requests for updating reinforcement learning hyperparameters, including request details, statuses, messages, and completion timestamps (Supplementary Table 6).
5. **RL Weights Table**: Contains information about the reinforcement learning model weights, including posterior means, variances, noise variance, and related metadata (Supplementary Table 7).
6. **RL Action Selection Table**: Records the actions selected by the algorithm for each participant, including decision indices, notification times, selected actions, probabilities, and rewards (Supplementary Table 8).
7. **Participant Action History Table**: Maintains a history of participant actions and responses, including decision indices, activity responses, app usage, cannabis use data, rewards, and timestamps (Supplementary Table 9).

Column Name	Data Type	Description
participant_id	String	Unique identifier for each participant
consent_start_date	DateTime	Start date of participant's consent
consent_end_date	DateTime	End date of participant's consent
rl_start_date	DateTime	Start date of reinforcement learning period
rl_end_date	DateTime	End date of reinforcement learning period

Supplementary Table 3: Participants Table

I MiWaves: Participant Verification Checks

The participant verification checks were performed to verify participants for the MiWaves clinical trial, and identify any fraudulent participants. The checks involved:

Column Name	Data Type	Description
participant_id	String	Foreign key referencing participants table
trial_phase	Enum	Current phase of the trial
morning_notif_time_start	Array(Integer)	Start times for morning notifications
evening_notif_time_start	Array(Integer)	Start times for evening notifications
current_decision_index	Integer	Index of the current decision point
current_time_of_day	Integer	Current time of day for the participant
trial_day	Integer	Current day in the trial

Supplementary Table 4: Participant Status Table

Column Name	Data Type	Description
policy_id	Integer	Unique identifier for the policy
update_time	DateTime	Timestamp of the last update
update_day_in_trial	Integer	Day in the trial when last updated
current_decision_time	Integer	Time of the current decision
current_day_in_trial	Integer	Current day in the trial

Supplementary Table 5: Algorithm Status Table

1. Limiting public information about eligibility criteria
2. Using password protected screening surveys
3. Editing screening survey settings to prevent multiple submissions from one respondent
4. Using a CAPTCHA verification at the beginning of the screening survey to protect against bots
5. Checking IP addresses associated with screening responses and ensure there are no duplicates
6. Completing identity checks with eligible screens (e.g. selfie checks, ID checks, social media checks)

Column Name	Data Type	Description
id	Integer	Unique request identifier
backup_location	String	Location of the backup file
request_timestamp	DateTime	Timestamp of the request
request_status	String	Status of the request
request_message	String	Message associated with the request
request_error_code	Integer	Error code if the request failed
completed_timestamp	DateTime	Timestamp when the request was completed

Supplementary Table 6: RL Hyperparameter Update Request Table

Column Name	Data Type	Description
id	Integer	Unique identifier for the RL weights record
policy_id	Integer	Identifier for the policy associated with the weights
update_timestamp	DateTime	Timestamp when the weights were updated
post_mean_array	Array(Float)	Array of posterior mean values
post_var_array	Array(Float)	Array of posterior variance values
post_tpop_mean_array	Array(Float)	Array of posterior population mean values
post_tpop_var_array	Array(Float)	Array of posterior population variance values
noise_var	Float	Variance of the noise in the model
random_eff_cov_array	Array(Float)	Covariance array for random effects
code_commit_id	String	Identifier for the code commit associated with the weights
data_pickle_file_path	String	Path to the data pickle file
participant_list	Array(String)	List of participants associated with the weights
hp_update_id	Integer	Identifier for the hyperparameter update request

Supplementary Table 7: RL Weights Table

Column Name	Data Type	Description
participant_id	String	Foreign key referencing participants table
participant_decision_idx	Integer	Index of the participant's decision
morning_notification_time	Array(Integer)	Times for morning notifications
evening_notification_time	Array(Integer)	Times for evening notifications
day_in_trial	Integer	Current day in the trial for the participant
action	Integer	Action selected by the algorithm
policy_id	Integer	Identifier for the policy used
seed	Integer	Seed used for random number generation
prior_ema_completion_time	String	Time taken to complete prior EMA
action_selection_timestamp	DateTime	Timestamp when action was selected
message_sent_notification_ts	String	Timestamp when message was sent
message_click_notification_ts	String	Timestamp when message was clicked
act_prob	Float	Probability of the selected action
cannabis_use	Array(Float)	Participant's cannabis use data
state_vector	Array(Float)	State vector for the participant
reward	Float	Reward received for the action
row_complete	Boolean	Indicates if the row is complete
rid	Integer	Foreign key referencing participant_action_history table

Supplementary Table 8: RL Action Selection Table

**Column Name**	**Data Type**	**Description**
index	Integer	Unique identifier for the participant action history record
participant_id	String	Foreign key referencing participants table
decision_idx	Integer	Index of the decision in the participant's history
finished_ema	Boolean	Indicates if the EMA was completed
activity_question_response	String	Participant's response to activity question
app_use_flag	Boolean	Indicates if the app was used
cannabis_use	Array(Float)	Participant's cannabis use data
reward	Float	Reward associated with the action
state	Array(Integer)	State array for the participant
action	Integer	Action taken by the participant
seed	Integer	Seed used for random number generation
act_prob	Float	Probability of the action taken
policy_id	Integer	Identifier for the policy used
timestamp	DateTime	Timestamp of the action

Supplementary Table 9: Participant Action History Table

J Monitoring Systems: Quantitative Data

Here we report quantitative data on red and yellow severity issues that occurred and the fallback methods that were executed during both trials.

J.1 Oralytics

Severity	Number of reported instances	Examples of issues caught
Red	0	N/A
Yellow	15	Fail to get app analytics data from backend controller

Supplementary Table 10: Number of issues detected during Oralytics deployment by red and yellow severity. Notice how green severity numbers are not reported because green severity issues represent inconsistencies or events that do not immediately affect the participant nor the RL algorithm but *must* be documented and passed to the data analyst to ensure valid post-deployment analyses.

Yellow Severity Issue	Number of Occurrences	Total Participants Impacted	Fallback Method
Y2: Endpoint response from app cannot be json-ified	8	8	(iii)
Y5: Action selection procedure failed	3	7	(ii)
Y7: RL Service and endpoints went down	4	94	(i)

Supplementary Table 11: Fallback methods executed during Oralytics deployment.

J.1.1 Oralytics Fallback Methods

We re-state the Oralytics fallback methods from the main paper for readability.

(i) if there were any communication issues between the backend controller and the app, intervention prompt from the most recent backup intervention schedule saved on the Oralytics app was delivered; and

(ii) if there were any issues in constructing the backup intervention schedule, then the binary action for each decision point was assigned with 0.5 probability

(iii) if issues arose with pulling or storing data (e.g., malformed data or recent data was not available), then the algorithm stored the data point, but did not update the JITAI decision rules using that data point.

J.2 MiWaves

Severity	Number of reported instances	Examples of issues caught
Red	3	Cloud computer ran out of memory, and both backend controller and RL algorithm crashed
Yellow	1	RL algorithm and backend controller had mismatch in number of active participants, and hence could not randomize certain active participants

Supplementary Table 12: Number of issues detected during MiWaves deployment by red and yellow severity. Notice how green severity numbers are not reported because green severity issues represent inconsistencies or events that do not immediately affect the participant nor the RL algorithm but *must* be documented and passed to the data analyst to ensure valid post-deployment analyses.

Yellow Severity Issue	Number of Occurrences	Total Participants Impacted	Fallback Method
Y206: Error trying to compute action by the RL algorithm	2	37	(i)

Supplementary Table 13: Fallback methods executed during MiWaves deployment.

J.2.1 MiWaves Fallback Methods

We re-state the MiWaves fallback methods from the main paper for readability.

(i) if there were any issues in requesting actions from the RL algorithm (e.g. communication errors, RL algorithm crashes etc.), then the binary action for each decision point was assigned with 0.5 probability by the backend controller

(ii) if issues arose while updating JITAI decision rules due to malformed data, the RL algorithm would not update the JITAI decision rules, and continue utilizing the last updated JITAI decision rules to select actions.

References

[1] Anna L. Trella, Kelly W. Zhang, Stephanie M. Carpenter, David Elashoff, Zara M. Greer, Inbal Nahum-Shani, Dennis Ruenger, Vivek Shetty, and Susan A. Murphy. Oralytics reinforcement learning algorithm, 2024.

[2] Inbal Nahum-Shani, Zara M Greer, Anna L Trella, Kelly W Zhang, Stephanie M Carpenter, Dennis Ruenger, David Elashoff, Susan A Murphy, and Vivek Shetty. Optimizing an adaptive digital oral health intervention for promoting oral self-care behaviors: Micro-randomized trial protocol. *Contemporary Clinical Trials*, page 107464, 2024.

[3] Anna L. Trella, Kelly W. Zhang, Inbal Nahum-Shani, Vivek Shetty, Finale Doshi-Velez, and Susan A. Murphy. Reward design for an online reinforcement learning algorithm supporting oral self-care. In Brian Williams, Yiling Chen, and Jennifer Neville, editors, *Thirty-Seventh AAAI Conference on Artificial Intelligence, AAAI 2023, Thirty-Fifth Conference on Innovative Applications of Artificial Intelligence, IAAI 2023, Thirteenth Symposium on Educational Advances in Artificial Intelligence, EAAI 2023, Washington, DC, USA, February 7-14, 2023*, pages 15724–15730. AAAI Press, 2023.

[4] Kelly W Zhang, Lucas Janson, and Susan A Murphy. Statistical inference after adaptive sampling for longitudinal data. *arXiv preprint arXiv:2202.07098*, 2022.

[5] Jiayu Yao, Emma Brunskill, Weiwei Pan, Susan Murphy, and Finale Doshi-Velez. Power constrained bandits. In *Machine Learning for Healthcare Conference*, pages 209–259. PMLR, 2021.

[6] Lara N Coughlin, Maya Campbell, Tiffany Wheeler, Chavez Rodriguez, Autumn Rae Florimbio, Susobhan Ghosh, Yongyi Guo, Pei-Yao Hung, Mark W Newman, Huijie Pan, et al. A mobile health intervention for emerging adults with regular cannabis use: A micro-randomized pilot trial design protocol. *Contemporary Clinical Trials*, page 107667, 2024.

[7] Susobhan Ghosh, Yongyi Guo, Pei-Yao Hung, Lara Coughlin, Erin Bonar, Inbal Nahum-Shani, Maureen Walton, and Susan Murphy. reBandit: Random Effects based Online RL algorithm for Reducing Cannabis Use. *arXiv preprint arXiv:2402.17739*, 2024.

[8] Susobhan Ghosh, Yongyi Guo, Pei-Yao Hung, Lara Coughlin, Erin Bonar, Inbal Nahum-Shani, Maureen Walton, and Susan Murphy. Miwaves reinforcement learning algorithm, 2024.

[9] Kristjan Greenewald, Ambuj Tewari, Susan Murphy, and Predag Klasnja. Action centered contextual bandits. *Advances in neural information processing systems*, 30, 2017.
